# DYNLT1 promotes mitochondrial metabolism to fuel breast cancer development by inhibiting ubiquitination degradation of VDAC1

**DOI:** 10.1186/s10020-023-00663-0

**Published:** 2023-06-06

**Authors:** Ling Huang, Bo Wei, Yuran Zhao, Xue Gong, Liming Chen

**Affiliations:** 1 Department of Biochemistry, School of Life Sciences, Nanjing Normal University, Nanjing, 210023 China; 2 Cancer Institute, School of Life Sciences, Nanjing Normal University, Nanjing, 210023 China; 3 Nanjing Maternal and Child Health Institute, Nanjing Maternity and Child Health Care Hospital, Women’s Hospital of Nanjing Medical University, Nanjing, 210004 China

**Keywords:** DYNLT1, VDAC1, Breast cancer, Mitochondrial metabolism, Protein stability

## Abstract

**Background:**

Mitochondrial metabolism has been proposed as an attractive target for breast cancer therapy. The discovery of new mechanisms underlying mitochondrial dysfunction will facilitate the development of new metabolic inhibitors to improve the clinical treatment of breast cancer patients. DYNLT1 (Dynein Light Chain Tctex-Type 1) is a key component of the motor complex that transports cellular cargo along microtubules in the cell, but whether and how DYNLT1 affects mitochondrial metabolism and breast cancer has not been reported.

**Methods:**

The expression levels of DYNLT1 were analyzed in clinical samples and a panel of cell lines. The role of DYNLT1 in breast cancer development was investigated using in vivo mouse models and in vitro cell assays, including CCK-8, plate cloning and transwell assay. The role of DYNLT1 in regulating mitochondrial metabolism in breast cancer development is examined by measuring mitochondrial membrane potential and ATP levels. To investigate the underlying molecular mechanism, many methods, including but not limited to Co-IP and ubiquitination assay were used.

**Results:**

First, we found that DYNLT1 was upregulated in breast tumors, especially in ER + and TNBC subtypes. DYNLT1 promotes the proliferation, migration, invasion and mitochondrial metabolism in breast cancer cells in vitro and breast tumor development in vivo. DYNLT1 colocalizes with voltage-dependent anion channel 1 (VDAC1) on mitochondria to regulate key metabolic and energy functions. Mechanistically, DYNLT1 stabilizes the voltage-dependent anion channel 1 (VDAC1) by hindering E3 ligase Parkin-mediated VDAC1 ubiquitination and degradation.

**Conclusion:**

Our data demonstrate that DYNLT1 promotes mitochondrial metabolism to fuel breast cancer development by inhibiting Parkin-mediated ubiquitination degradation of VDAC1. This study suggests that mitochondrial metabolism can be exploited by targeting the DYNLT1-Parkin-VDAC1 axis to improve the ability of metabolic inhibitors to suppress cancers with limited treatment options, such as triple-negative breast cancer (TNBC).

**Supplementary Information:**

The online version contains supplementary material available at 10.1186/s10020-023-00663-0.

## Introduction

Breast cancer, is the most common human cancer, with an estimated 2.3 million new cases in 2020 (Sung et al. 2021). In recent decades, targeted therapy and molecular targeting of multiple signaling pathways to interfere with multiple oncogenic pathways has became a common and evolving therapeutic approach for breast cancer (Tsimberidou 2015).

Metabolic remodeling is one of the hallmarks of cancer, among which the Warburg effect is an important sign of abnormal metabolism in cancer. Although the understanding of Warburg effect has focused most of the research on cancer metabolism on aerobic glycolysis, abnormal metabolic processes such as respiration and energy conversion involving mitochondria still play an important role (Zong et al. 2016; Vasan et al. 2020). Inhibitors targeting multiple pathways involved in mitochondrial metabolism, including inhibition of cellular components involved in mitochondrial synthesis, reduction of metabolite accumulation or blocking energy synthesis in mitochondria, have played a therapeutic role in a variety of cancers. For example, Devimistat (CPI-613), a lipoic acid analog that inhibits pyruvate dehydrogenase (PDH) and ɑ-ketoglutarate dehydrogenase and disrupts mitochondrial metabolism, has shown antitumor activity in non-small cell lung cancer and pancreatic tumors (Vasan et al. 2020). IACS-010759, a mitochondrial complex I inhibitor, has been shown to inhibit tumor growth and induce apoptosis in a tumor model dependent on oxidative phosphorylation (OXPHOS), and can be used in combination with PD-1 monoclonal antibody to treat non-small cell lung cancer (Vasan et al. 2020). Therefore, targeting mitochondrial metabolism remains a potential target for cancer therapy, and the key is to discover and elucidate the genes that cause the disorder of mitochondrial metabolism in tumors. However, the underlying mechanism in breast cancer is not completely understood.

DYNLT1 is a member of the cytoplasmic dynein family, which is responsible for material transport along microtubules (Machado et al. 2003; Allan 2011). In addition, DYNLT1 can regulate the length of primary cilia and interact with dynein protein to promote ciliary protein transport (King 2000; Pfister et al. 2005; Allan 2011). DYNLT1 is also involved in the assembly of male sperm flagellin and plays a key role in sperm development and its abnormal expression is closely associated with male infertility (Indu et al. 2015; Elzeiny et al. 2019). DYNLT1 in breast cancer remains largely unknown. Based on our previous analysis of the TCGA dataset, we found that DYNLT1 was significantly highly expressed in breast cancer and correlated with prognosis. Therefore, we speculated whether DYNLT1 can be used as a novel target to regulate breast cancer development and wanted to further explore the mechanism behind it.

In our study, we report that DYNLT1 is an important regulator in mitochondrial metabolism for the development and progression of breast cancer. DYNLT1 increases the mitochondrial membrane potential by stabilizing voltage-dependent anion channel 1 (VDAC1), resulting into the increased production of ATP by mitochondria. The ability of DYNLT1 to stabilize VDAC1 is controlled by the ubiquitin proteolytic pathway, where DYNLT1 inhibits E3 ligase Parkin from binding and ubiquitinating VDAC1. Taken together, this study demonstrates DYNLT1-Parkin-VDAC1 axis to be a new mechanism that underlies the mitochondrial metabolism for breast cancer development, improving our mechanistic understanding on the mitochondrial metabolism in breast cancer and providing new targets for future development of mitochondrial inhibitors to suppress breast cancer.

## Materials and methods

### Cell culture

All cells involved in this article including MCF10A, MCF-7, T47D, SKBR3, MDA-MB-468, MDA-MB-231, 4T1 and 293T were obtained from ATCC. All cells were cultured in medium and supplemented with 10% fetal bovine serum and 1% penicillin/streptomycin. Cells were incubated at 37 °C in humidified air containing 5% CO_2_.

### Plasmid transfection

The pcDNA3.1-His-Ubiquitin, pcDNA3.1-His, CMV-3×Flag-DYNLT1, CMV-3×Flag, pcDNA3.0-HA or pcDNA3.0-HA-VDAC1 plasmid was transfected into 293T, MCF-7 or MDA-MB-468 cells using Lipofectamine 2000 (Invitrogen) according to the product brochures. Full-length DYNLT1 and VDAC1 cDNA was generated by PCR with specific primers and then ligated into CMV-3×Flag and pcDNA3.0-HA vector, respectively. The transfected cells were maintained for 48 h and then subjected to subsequent in vitro cell phenotype, WB, RT-qPCR or ATP level measurement experiments.

### Lentiviral shRNA knockdown and in vitro drug treatment

The pLKO.1-based lentiviral shRNA technique was used to construct the MCF-7, MDA-MB-468 and 4T1 cells with stably expressed DYNLT1 shRNA or nontargeted control shRNA. The shRNA plasmid, along with packaging plasmids, was transfected into the 293T cells to produce lentiviral particles. The MCF-7, MDA-MB-468 and 4T1 cells were infected with lentiviruses expressing the shRNA constructs. Stable MCF-7, MDA-MB-468 and 4T1 cells were generated with 2 µg/ml, 0.5 µg/ml or 3 µg/ml puromycin (Invivogen) for 10 days. The shRNA sequences are shown as follow: Human-DYNLT1 shRNA-1: 5′- GAGGCTATAGAAAGCGCAATT − 3′; Human-DYNLT1 shRNA-2: 5′- GAGCTGGATTACACACAGCAA − 3′; Mouse-DYNLT1 shRNA-1: 5′- TGAAGTGAGCAGCATTGTAAA − 3′; Mouse-DYNLT1 shRNA-2: 5′- GTCAACCAGTGGACCACTAAT − 3.

For in vitro drug treatment, 7 × 10^4^ MCF-7 or MDA-MB-468 cells with stably expressed the DYNLT1 shRNA or nontargeted control shRNA were seeded in 6-well plates and cultured overnight. 100 µg/ml CHX (Cycloheximide) or 20 µM MG132 (selleck) was added to the cell culture medium, and the cell pellet was collected after 48 h for subsequent WB experiments.

### Cell counting Kit-8 (CCK-8) assay

For cell proliferation, MCF-7 and MDA-MB-468 cells were seeded into 96-well plates (1 × 10^3^ cells/well) after knockdown or overexpression of DYNLT1 treatment, 10 µl CCK-8 (Dojindo) solution was added into each well and the cells incubated at 37 °C for 2 h. Then reading the absorbance at 450 nm using a Bio-Rad iMark plate reader.

### Transwell assay

Polyester membrane cell embedding dishes with 24-well plates (insert: 8.0 μm, JET, Guangzhou, China) were used to examine the migration or invasion abilities of breast cancer cells. Cells were seeded into 24-well plates at a density of 2 × 10^4^ or 4 × 10^4^ cells/well with serum-free cell culture into the upper chamber. For the invasion assay, matrigel was prelaid before inoculating cells into 24-well plates. Cell culture containing 10% FBS was placed in the lower chamber. After 16–24 h incubation, cells that had invaded the lower chamber were fixed with methanol for 20 min and stained with 0.5% Crystal Violet for 20 min. Finally, the cell migration or invasion was recorded by microscopy. Three randomly selected fields of view were observed and the number of invaded cells were counted under the microscope. This experiment was repeated three times.

### Plate clone formation assay

The MCF-7 or MDA-MB-468 cells with knockdown or overexpressed DYNLT1 were plated into 6-well plates at a density of 500 or 10,000 cells per well with complete medium. Cells were cultured for 14 days and then fixed with 4% PFA and stained with Giemsa stain (Leagene) and then the number of the clones were counted and analyzed.

### Western blot and antibodies

Total protein was extracted from collected cells and lysed in ice-cold RIPA buffer (Beyotime) for 15 min on ice with a protease inhibitor. Protein lysates were separated via SDS-PAGE and transferred onto PVDF membranes (Millipore) incubated with indicated primary antibodies and then secondary antibodies conjugated HRP. The ECL kit (Millipore) was used for detection of targeted signals. Antibodies used in western blot were as follows: GAPDH (ABclonal), DYNLT1 (Abcam), VDAC1 (Proteintech), Parkin (ABclonal), Flag (ABclonal), HA (ABclonal).

### RNA extraction and RT-qPCR

Total RNAs were extracted from cells using the Total RNA Isolation Kit (BEI-BEI BIOTECH) based on the manufacturer’s instructions and quantified with the NanoDrop spectrophotometer. For RT-qPCR, cDNAs were synthesized with the HiScript II Q RT SuperMix for qPCR (Vazyme) and PCR reactions were performed with ChamQ SYBR qPCR Master Mix (Vazyme). The primers for RT-qPCR are as follows: GAPDH forward: 5’-AGAAGGCTGGGGCTCATTTG-3’, GAPDH reverse: 5’-AGGGGCCATCCACAGTCTTC-3; DYNLT1 forward: GTGGTAACGCTTATCAACACAGC, DYNLT1 reverse: GCTTGGTGAGTTGGCTTAAAGT; VDAC1 forward: GCAAAATCCCGAGTGACCCAGA, VDAC1 reverse: TCCAGGCAAGATTGACAGCGGT.

### Mitochondrial membrane potential

The mitochondrial membrane potential of MCF-7 and MDA-MB-468 cells with DYNLT1 knockdown was evaluated with a mitochondrial membrane potential assay kit containing JC-1 (Beyotime). Briefly, cells were harvested and stained with JC-1 for 20 min at 37 °C. After washing twice with PBS, the mitochondrial membrane potential was measured under a fluorescence microscope (Leica). To measure the red or green fluorescence intensity, the excitation wavelength was read at 525 or 485 nm and the emission wavelength was read at 590 or 525 nm.

### Cellular ATP measurement

Cellular ATP level was measured according to an ATP assay kit (Beyotime). Briefly, Culture supernatant was removed and followed by addition of 100 µl of ATP lysis buffer to each well. After centrifugation at 12,000 g at 4 °C for 5 min, the supernatant was collected. And 100 µl of reaction solution was added to each well in a white 96-well plate. Then 20 µl sample or standard were added to the corresponding wells. The plate was measured using a multifunctional microplate reader (TECAN) and measured absorbance at 525 nm. Standard curves were drawn according to the instructions and ATP content was calculated according to the standard curves. The protein level was determined using a BCA protein assay kit (Thermo Scientific, Rockford, IL). The ATP level was normalized to the protein concentration of each sample.

### Immunofluorescence analysis

Cells were seeded into 24-well plates at a density of 5000 cells/well. 200 nM Mito-Tracker Red CMXRos (Beyotime) was added into cell culture and incubated for 30 min to label the mitochondria. After removing the media, they were washed with PBS, and fixed for 20 min in 4% PFA. Cells were permeabilized for 10 min with 0.5% Triton X-100 and blocked for 1 h with 1% BSA at room temperature. After washing with PBST (PBS containing with 0.5% Triton X-100), the cells were incubated overnight with either rabbit anti-DYNLT1 antibody (Abcam) or mouse anti-VDAC1 antibody (Proteintech) at 4℃, and then washed with PBST. Cells are incubated with fluorescein isothiocyanate-conjugated goat anti-rabbit IgG antibody or anti-mouse IgG antibody for 1 h at 37℃ in the dark. Cells were counterstained with DAPI nuclear staining dye. After extensive washing with PBS, the slides were mounted in a drop of mounting medium to reduce photobleaching.

### Separation of mitochondrial and non-mitochondrial fractions

Mitochondrial fractions were separated according to a commercial kit (Beyotime). Briefly, after collecting the cell precipitate, 1 ml mitochondrial isolation solution was added on ice for 10 min, followed by homogenization for 2 min. Then, cells were centrifuged at 600 g for 10 min at 4 ℃. The supernatants were collected and further centrifuged at 11,000 g for 10 min at 4 ℃. The precipitation is mitochondrial fraction and the supernatant is non-mitochondrial fraction, and then the protein was cleaved and extracted respectively for subsequent WB verification.

### Data analysis

Transcriptome data and clinical information of breast cancer were downloaded from the TCGA (The Cancer Genome Atlas) database (https://portal.gdc.cancer.gov/). DYNLT1 expression levels in breast cancer samples of different subtypes and significance were calculated. Visualize Kaplan Meier survival curve of patients under different gene expression groups by using survival R package. Multivariate cox regression analysis was used to calculate the contribution of genes to patient survival after age adjustment. The GO and KEGG enrichment analysis was performed to reveal the enriched biological processes, cellular components, molecular functions or signaling pathways using the clusterProfiler R software package (Yu et al. 2012). The top 500 genes significantly associated with DYNLT1 are listed in Table S1 and pearson correlation coefficient is calculated.

The spatial transcriptome data of breast cancer is from the study of Wu et al. (Wu et al. 2021) and download from https://zenodo.org/record/4739739#.YzaiA3ZBxD9. Spatial transcriptome data were integrated using Seurat. Those spots with more than 20% mitochondria and less than 500 genes were discarded. SCTransform function is used to standardize the data of spatial transcriptome and single cell transcriptome. Use FindTransferAnchors and TransferData functions to integrate spatial transcriptome data and single cell transcriptome. Use TransferData to map the cell type of a single cell to a spatial location.

Each experiment was performed three to six times. Statistical analysis was performed using GraphPad Prism 7. The t-test was used for statistic quantification (*, p < 0.05; **, p < 0.01; ***, p < 0.001; ****, p < 0.0001; ns, not significant).

## Results

### DYNLT1 is highly expressed in breast cancer and promotes breast cancer development

**Fig. 1:**
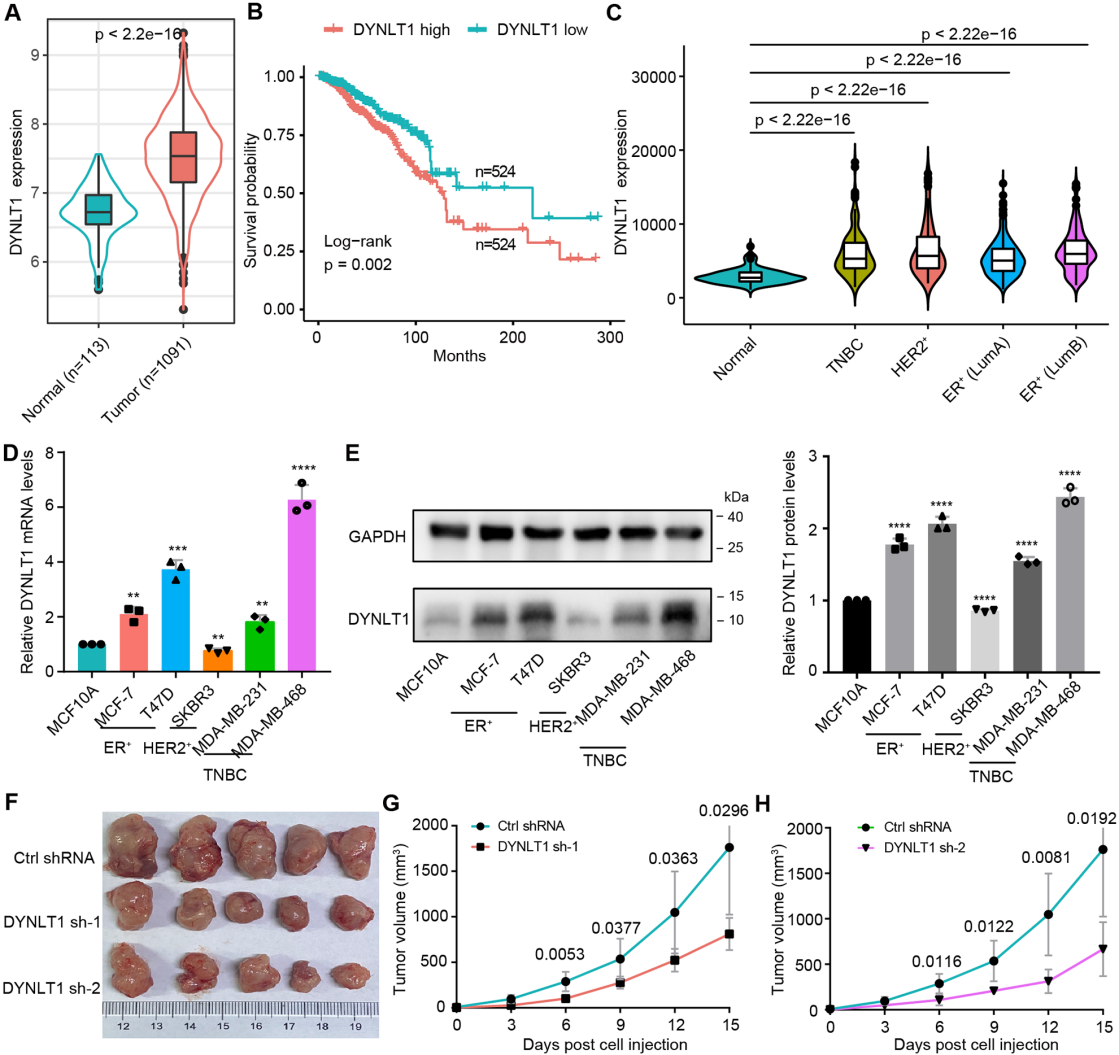
DYNLT1 is a novel therapeutic target for breast cancer and promotes breast cancer development **A** Differential expression of DYNLT1 in breast tumor tissues and normal tissues. **B** Survival rate of breast cancer patients in high and low DYNLT1 expression group. **C** Analysis of DYNLT1 expression in different subtypes of breast cancer. **D** Relative mRNA levels of DYNLT1 in different subtypes of breast cancer cells. **E** Protein levels of DYNLT1 in different subtypes of breast cancer cells. On the right are statistics of the protein gray value ratio of DYNLT1/GAPDH. **F-H** Examination on tumors formed by stable DYNLT1 knockdown 4T1 cells in Balb/c mice: (**F**) Representative pictures of tumors derived from mice; (**G, H**) Tumor growth from mice with indicated treatment. **, p < 0.01; ***, p < 0.001; ****, p < 0.0001. All experiments were performed at least three replicates

First, we analyzed breast cancer data from TCGA using bioinformatics methods and found that DYNLT1 was differentially expressed in breast cancer and was significantly higher in tumor tissues than in normal tissues (Fig. 1A). Survival analysis showed that breast cancer patients with high DYNLT1 expression had a poor prognosis (Fig. 1B). Clinically, using estrogen receptor (ER), progesterone receptor (PR) and human epidermal growth factor receptor 2 (HER2) as markers, breast cancer patients are mainly classified into ER+, HER2 + and triple-negative breast cancer (TNBC) subtypes. In the classification analysis of breast cancer, we found that the expression of DYNLT1 in various subtypes of breast cancer tissues was higher than in normal tissues (Fig. 1C). Then, we detected the expression of DYNLT1 in different subtypes of breast cancer cell lines by RT-qPCR and western blot (WB) assay, respectively, and found that DYNLT1 was highly expressed in ER + breast cancer cells, including MCF-7 and T47D, and TNBC cells, including MDA-MB-468 and MDA-MB-231 (Fig. 1D and E). We then knocked down the expression of DYNLT1 in 4T1 breast cancer cells and performed in vivo mouse tumorigenesis assays. Western blot and RT-qPCR results showed the successful knockdown of DYNLT1 expression in 4T1 cells (Fig. S1A). Our study showed that knockdown of DYNLT1 significantly inhibited the growth of mouse breast cancer (Fig. 1F-H). These results confirmed that DYNLT1 is highly expressed in breast cancer, including but not limited to TNBC subtypes, suggesting that DYNLT1 may be a new therapeutic target for multiple subtypes of breast cancer.

### DYNLT1 promotes proliferation, clone formation, migration and invasion of breast cancer cells

**Fig. 2:**
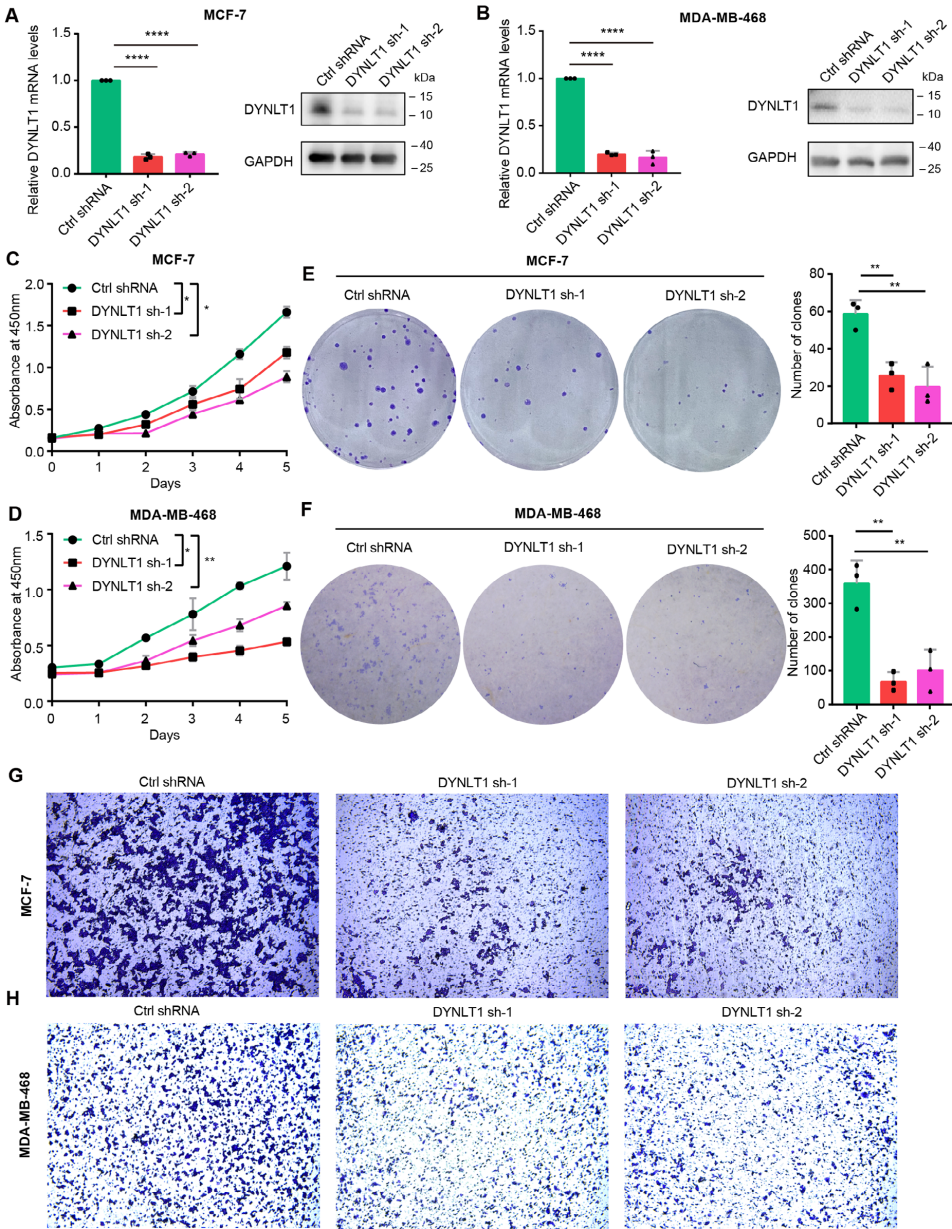
Low expression of DYNLT1 inhibits the proliferation, clone formation, and migration of breast cancer **A-B** RT-qPCR and Western blot showed the successful construction of stable DYNLT1 knockdown MCF-7 (A) or MDA-MB-468 (B) breast cancer cells. **C-D** The proliferation of stable DYNLT1 knockdown MCF-7 (C) or MDA-MB-468 (D) breast cancer cells by CCK8 assay. **E-F** The ability of clone formation of stable DYNLT1 knockdown MCF-7 (E) or MDA-MB-468 (F) breast cancer cells. The graph on the right shows the statistics of the number of clones. **G-H** The migration of stable DYNLT1 knockdown MCF-7 (G) or MDA-MB-468 (H) breast cancer cells. *, p < 0.05; **, p < 0.01; ****, p < 0.0001. All experiments were performed at least three replicates

**Fig. 3:**
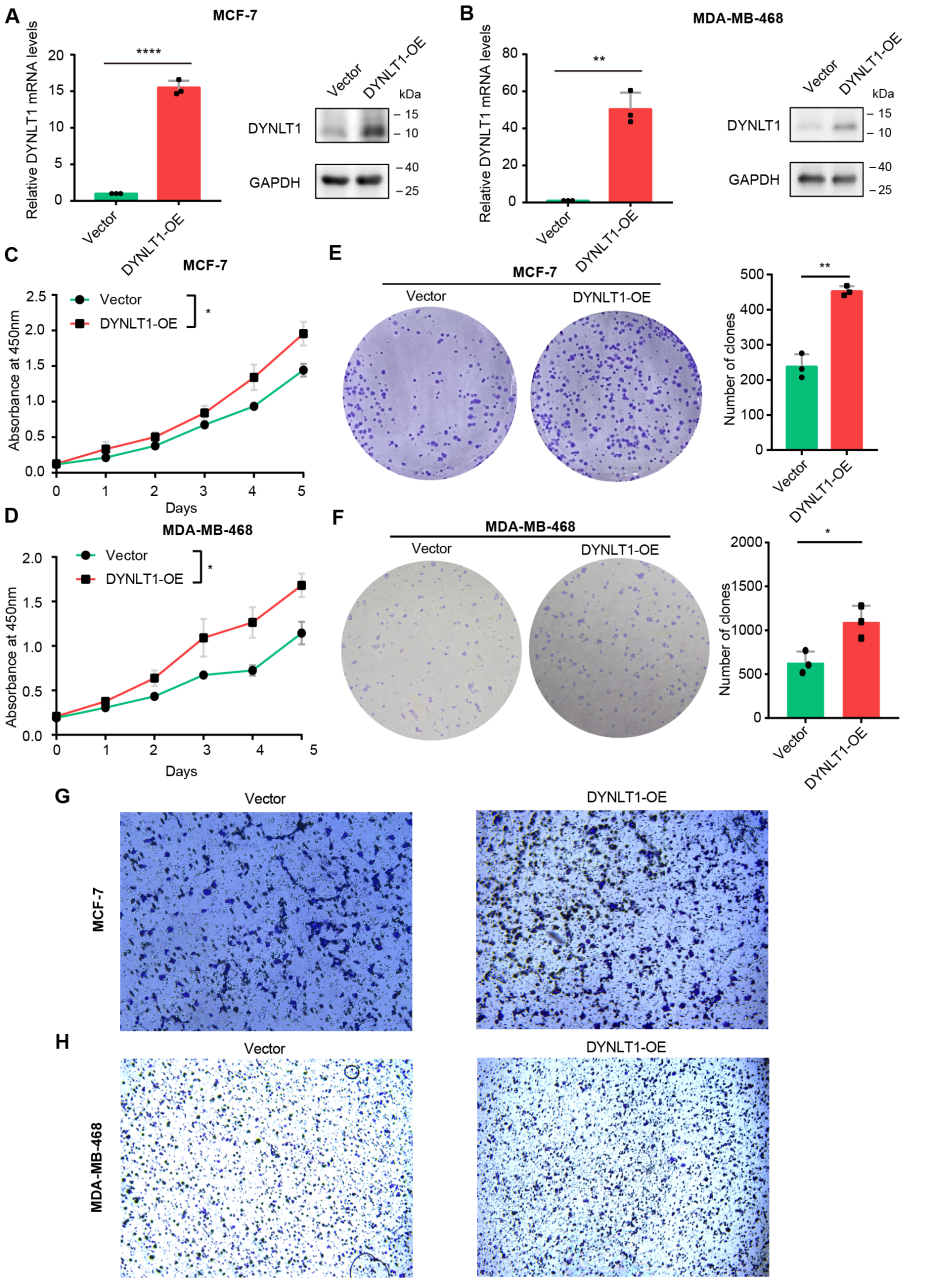
High expression of DYNLT1 promotes the proliferation, clone formation, and migration of breast cancer **A-B** RT-qPCR and Western blot showed the successful overexpression of DYNLT1 in MCF-7 (**A**) or MDA-MB-468 (**B**) breast cancer cells. **C-D** The proliferation after overexpression of DYNLT1 in MCF-7 (**C**) or MDA-MB-468 (**D**) breast cancer cells. **E-F** The ability of clone formation after overexpression of DYNLT1 in MCF-7 (**E**) or MDA-MB-468 (**F**) breast cancer cells. The graph on the right shows the statistics of the number of clones. **G-H** The migration after overexpression of DYNLT1 in MCF-7 (**G**) or MDA-MB-468 (**H**) breast cancer cells. *, p < 0.05; **, p < 0.01; ****, p < 0.0001. All experiments were performed at least three replicates

Next, we wanted to further explore the function of DYNLT1 in breast cancer. We constructed DYNLT1 stable knockdown cell lines for subsequent studies using ER + MCF-7 and triple negative MDA-MB-468 breast cancer cells, respectively. The successful construction of the cell lines was verified by RT-qPCR and WB, respectively (Fig. 2A and B). Then, we examined the effect of DYNLT1 knockdown on the proliferation, clone formation, migration and invasion of breast cancer cells. Inhibition of DYNLT1 expression significantly reduced the proliferation and clone formation ability in MCF-7 and MDA-MB-468 cells (Fig. 2C-F). According to the transwell assay, we found that DYNLT1 knockdown also significantly inhibited the migration and invasion ability of ER + and TNBC cells (Fig. 2G-H and Fig. S1B-C). Consistent with these findings, when we overexpressed DYNLT1 levels in MCF-7 and MDA-MB-468 breast cancer cells (Fig. 3A and B), the overexpression of DYNLT1 inversely enhanced cell proliferation, clone formation, migration and invasion (Fig. 3C-H and Fig. S1D-E). Next, we sought to further confirm the oncogenic ability of DYNLT1 in breast cancer. We overexpressed DYNLT1 in MCF-7 or MDA-MB-468 cells with stable DYNLT1 knockdown and re-examined a series of cell phenotypes. We found that overexpression of DYNLT1 restored the inhibition of cell proliferation, clone formation, migration and invasion in stable DYNLT1 knockdown MCF-7 or MDA-MB-468 breast cancer cells (Fig. S2). Together, these results collectively suggest that DYNLT1 promotes breast cancer development.

### DYNLT1 regulates mitochondrial metabolism in breast cancer

**Fig. 4:**
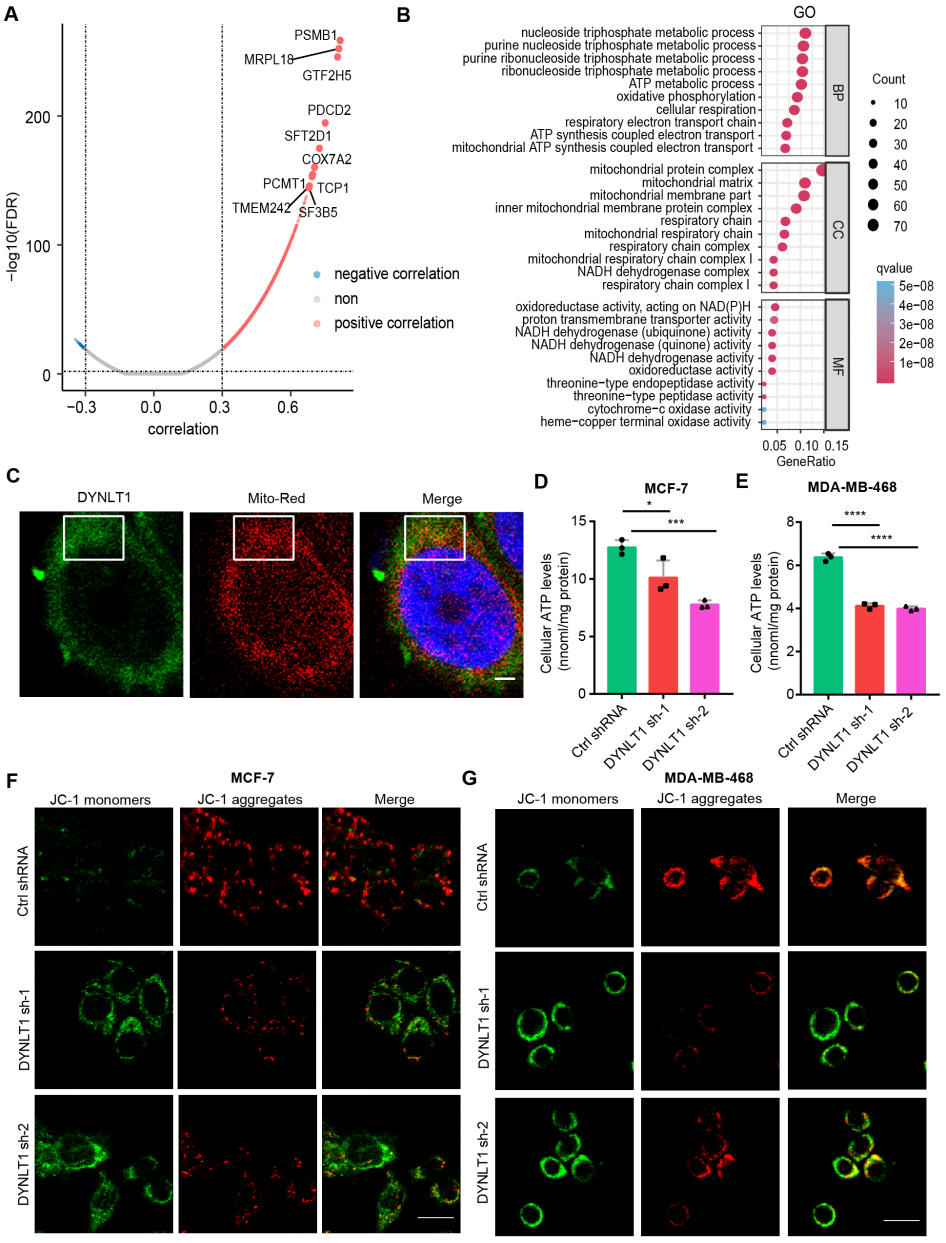
DYNLT1 regulates ATP levels and mitochondrial membrane potential in breast cancer cells **A** A volcano plot represents the genes correlated with DYNLT1: genes with significant negative correlation and positive correlation were navy and red respectively and genes with no significant correlation are colored gray. **B** Gene Ontology (GO) analysis revealed the enriched biological process (BPs), cellular components (CCs) and molecular functions (MFs) involved in top 500 genes that significantly correlated with DYNLT1. **C** Immunofluorescence results showed that DYNLT1 was co-localized with mitochondria. DYNLT1 (green), mitochondria (Red), nuclei (bule). Scale bar represents 2 μm. **D-E** The cellular ATP levels in stable DYNLT1 knockdown MCF-7 (D) or MDA-MB-468 (E) breast cancer cells. **F-G** Mitochondrial membrane potential in stable DYNLT1 knockdown MCF-7 (F) or MDA-MB-468 (G) breast cancer cells. *, p < 0.05; ***, p < 0.0001; ****, p < 0.0001. Scale bar represents 10 μm. All experiments were performed at least three replicates

As described above, in vitro and in vivo data demonstrate the oncogenic role of DYNLT1 in breast cancer development. To explore the underlying mechanism, firstly, we analyzed the genes significantly associated with DYNLT1 in breast cancer using TCGA data and plotted a volcano map of genes significantly positively or negatively associated with DYNLT1 based on correlation (Fig. 4A). According to the absolute value of the correlation coefficients, we obtained the top 500 genes with significant correlation, including PSMB1, MRPL18, GTF2H5, PDCD2, SFT2D1, COX7A2, PCMT1, TCP1, TMEM242 and SF3B5, and so on. Interestingly, all the top 500 genes were significantly positively correlated with DYNLT1 expression and most of the 500 genes significantly positively correlated with DYNLT1 were associated with poor prognosis of breast cancer, further suggesting the oncogenic effect of DYNLT1 (Table S1 and Fig. S1F). Then, we performed GO (gene ontology) and KEGG (Kyoto Encyclopedia of Genes and Genomes) pathway analysis on these top 500 genes to explore their biological functions or cell components. Biological process showed that the genes significantly positively correlated with DYNLT1 were significantly involved in biological functions related to ATP metabolic process and mitochondrial ATP synthesis coupled electron transport. Moreover, cell components and molecular functions showed that mitochondrial membrane proteins, respiratory chain and NADH dehydrogenase were significantly enriched (Fig. 4B). In addition, KEGG analysis was also enriched in pathways related to oxidative phosphorylation, proteasome, ribosome, and various diseases (Fig. S1G). To date, a large number of literature has shown that mitochondria play a key role in tumorigenesis, in which abnormal accumulation of mitochondrial metabolites such as ATP and transition of mitochondrial permeability can significantly promote malignant transformation of tumors, including breast cancer (Zong et al. 2016; Porporato et al. 2018). These findings suggest that DYNLT1 can participate in mitochondrial metabolism. To test this idea, we performed immunofluorescence staining to label DYNLT1 and mitochondria respectively, and the results showed that DYNLT1 was co-localized with the mitochondria in breast cancer cells (Fig. 4C). Then, we isolated the mitochondria in the cell, extracted the proteins in them, and found that DYNLT1 was significantly enriched in mitochondria, compared with the non-mitochondria by WB (Fig. S1H). Furthermore, when we explored the effect of DYNLT1 on the ATP levels in the mitochondrial metabolism of breast cancer, we found that the cellular ATP levels were significantly decreased and increased after DYNLT1 knockdown and overexpression, respectively, in breast cancer cells (Fig. 4D and E, Fig. S1I). These results indicated that DYNLT1 promoted mitochondrial metabolism. The mitochondrial membrane potential (MMP), which reflects the mitochondrial permeability transition (MPT), measures mitochondrial metabolism by using JC-1 (Huser and Blatter 1999). JC-1 is a fluorescent probe that aggregates in the matrix of mitochondria with high MMP to generate red fluorescence from green fluorescence by forming a polymer (aggregates). The results showed that using JC-1, DYNLT1 knockdown cells showed more low potential green fluorescence compared to the control shRNA cells with high potential red fluorescence (Fig. 4F and G). These data suggest that knockdown of DNYLT1 leads to a shift in mitochondrial permeability, resulting in decreased intracellular ATP levels. Together, these results demonstrate that DYNLT1 promotes mitochondrial metabolism by localizing to the mitochondria of breast cancer cells.

### DYNLT1 promotes mitochondrial metabolism by binding to VDAC1

**Fig. 5:**
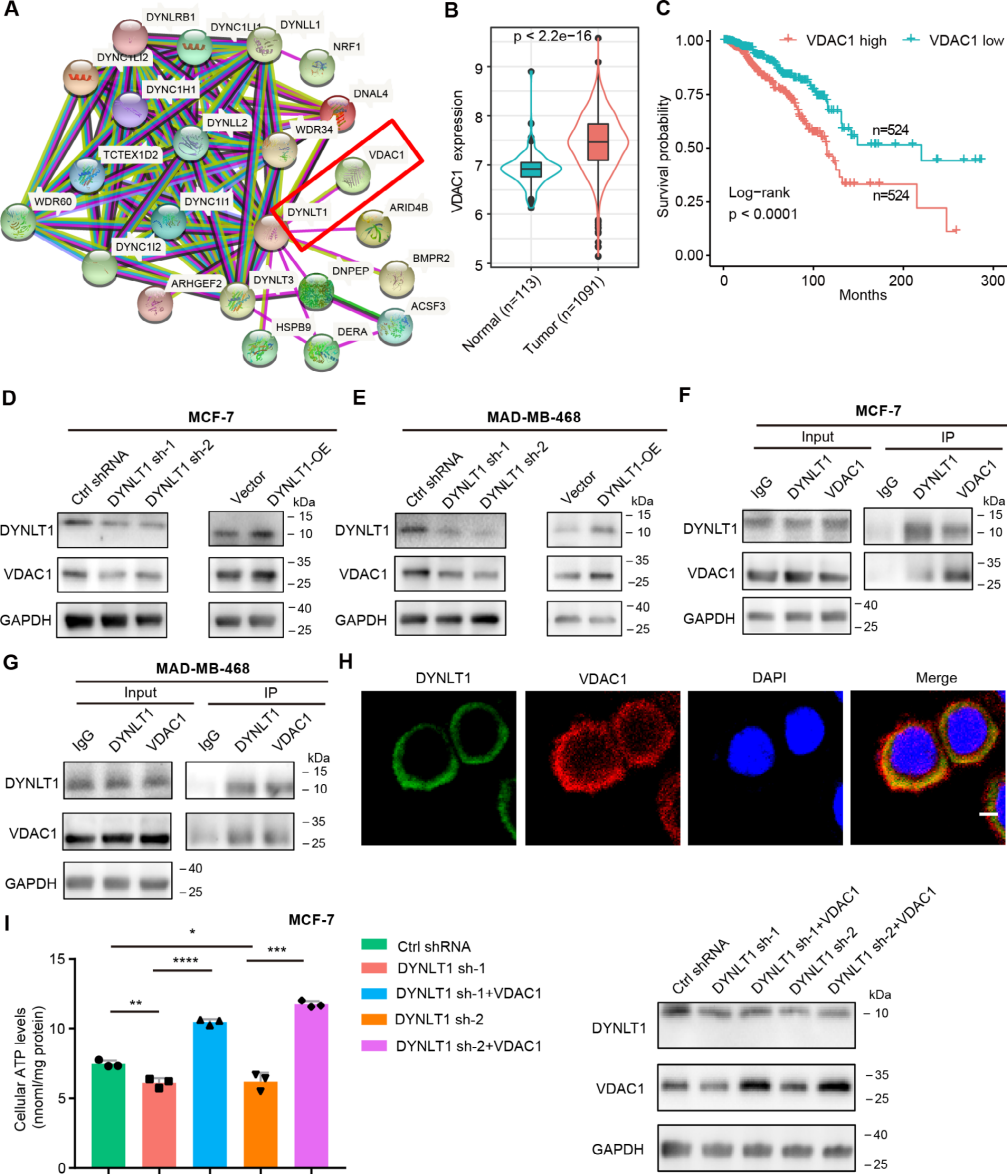
DYNLT1 colocalizes with VDAC1 in mitochondria to promote mitochondrial metabolism **A** Protein interaction analysis from STRING database. **B** Differential expression of VDAC1 in breast tumor tissues and normal tissues. **C** Survival rate of breast cancer patients in high and low VDAC1 expression group. **D-E** WB results showed that protein expression of GAPDH, DYNLT1 and VDAC1 in MCF-7 (**D**) or MDA-MB-468 (**E**) breast cancer cells with indicated treatment. **F-G** Co-IP results showed that DYNLT1 can interact with VDAC1 in MCF-7 (**F**) or MDA-MB-468 (**G**) breast cancer cells. **H** Immunofluorescence results showed that DYNLT1 was co-localized with VDAC1. DYNLT1 (green), VDAC1 (Red), DAPI (bule). Scale bar represents 2 μm. **I** The cellular ATP levels with indicated treatment in MCF-7 cells. The figure on the right shows the protein expressions of GAPDH, DYNLT1 and VDAC1 with indicated treatment. *, p < 0.05; **, p < 0.01; ***, p < 0.001, ****, p < 0.0001. All experiments were performed at least three replicates

Next, we wanted to further explore how DYNLT1 affects mitochondrial metabolism in breast cancer cells. The results of GO analysis suggested that the expression of DYNLT1 was positively correlated with the expression of mitochondrial membrane proteins. By analyzing the proteins that interact with DYNLT1 using the STRING database, we found that DYNLT1 has many interacting proteins, including voltage-dependent anion channel 1 (VDAC1) (Fig. 5A). VDAC1 is a major component of the mitochondrial outer membrane and regulates the entry and exit of metabolites and ATP across the mitochondrial outer membrane in mitochondrial metabolism (Heslop et al. 2021). Correlation analysis showed that VDAC1 was positively correlated with DYNLT1 (Fig. S3A). VDAC1 was also significantly overexpressed in breast cancer and the elevated expression of VDAC1 was also associated with poor prognosis in breast cancer (Fig. 5B and C). These findings led us to further investigate whether DYNLT1 contributes to mitochondrial metabolism in breast cancer by regulating VDAC1. After knockdown or overexpression of DYNLT1, the mRNA level of VDAC1 was not regulated by DYNLT1, while the protein level of VDAC1 was significantly inhibited or promoted (Fig. S3B and C, Fig. 5D and E). In addition, Co-IP results showed a significant endogenous interaction between DYNLT1 and VDAC1 in both MCF-7 and MDA-MB-468 breast cancer cells (Fig. 5F and G), further suggesting that VDAC1 protein is regulated by DYNLT1. As mentioned above, DYNLT1 localizes to mitochondria (Fig. 4C), and VDAC1 is one of the outer membrane proteins of mitochondria. We further confirmed the co-localization of DYNLT1 and VDAC1 by immunofluorescence (Fig. 5H). Moreover, spatial transcriptome data from clinical breast cancer samples also revealed extensive spatial colocalization of DLYNT1 and VDAC1 in cancer epithelial cells (Fig. S3D and E). These results suggest that DYNLT1 regulates the expression of VDAC1 at the protein level by interacting with VDAC1. When we overexpressed VDAC1 in DYNLT1 knockdown breast cancer cells and detected the changes in cellular ATP levels, we found that the reduced ATP levels after DYNLT1 knockdown was significantly increased after overexpression of VDAC1 (Fig. 5I and Fig. S3F), and WB results showed VDAC1 protein levels after overexpression in DYNLT1 knockdown cells. These results suggest that DYNLT1 colocalizes with VDAC1 on mitochondria and regulates VDAC1 at the protein level to promote mitochondrial metabolism in breast cancer cells.

### DYNLT1 inhibits VDAC1 degradation by hindering E3 ubiquitination ligase parkin from ubiquitinating VDAC1

**Fig. 6:**
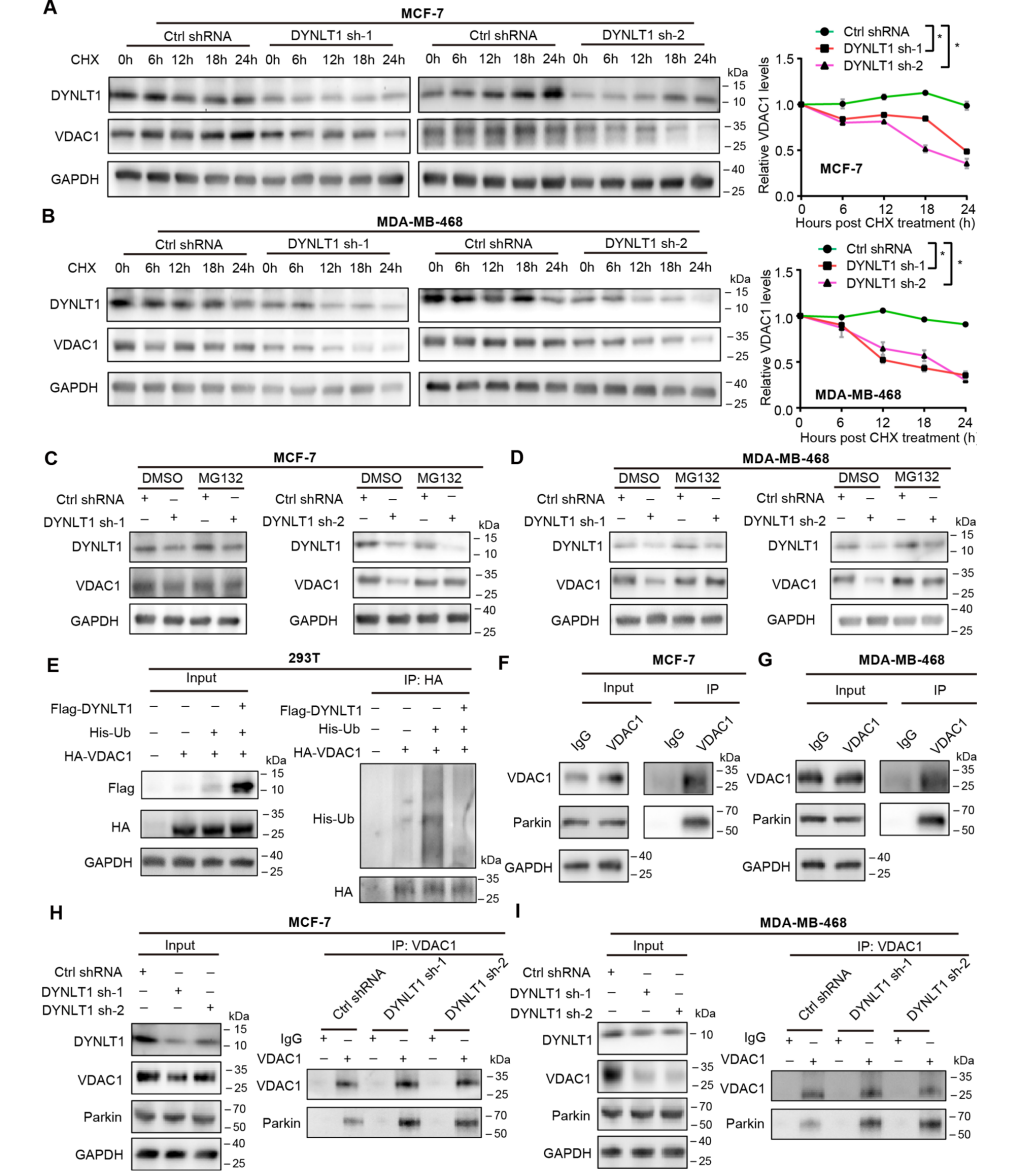
DYNLT1 antagonizes the interaction between Parkin and VDAC1 and stabilizing the expression of VDAC1 **A-B** WB results showed that DYNLT1 knockdown affected the protein stability of VDAC1. Line plots showed the relative protein levels of VDAC1 after DYNLT1 knockdown. (**A**): MCF-7 DYNLT1 knockdown cell; (**B**): MDA-MB-468 DYNLT1 knockdown cell. **C-D** Protein levels of DYNLT1, VDAC1 and GAPDH in stable DYNLT1 knockdown MCF-7 (**C**) or MDA-MB-468 (**D**) breast cancer cells treated with MG132. **E** Ubiquitination experiments showed that exogenous DYNLT1 overexpression in 293T cells significantly inhibited the ubiquitination degradation of VDAC1. **F-G** Co-IP results showed that Parkin can interact with VDAC1 in MCF-7 (**F**) or MDA-MB-468 (**G**) breast cancer cells. **H-I** Co-IP results showed that inhibition of DYNLT1 strength the interaction between Parkin and VDAC1 in stable DYNLT1 knockdown MCF-7 (**H**) or MDA-MB-468 (**I**) breast cancer cells. *, p < 0.05. All experiments were performed at least three replicates

We then investigated the specific mechanism by which DYNLT1 regulates VDAC1 at the protein level. We treated DYNLT1 knockdown cells with cycloheximide (CHX) to investigate whether inhibition of DYNLT1 could affect the protein stability of VDAC1. The WB results showed that the degradation rate of VDAC1 was significantly accelerated after DYNLT1 knockdown (Fig. 6A and B). It is well known that the ubiquitin-proteasome pathway is an important way to regulate protein stability. Therefore, when we treated DYNLT1 knockdown cells with MG132, a proteasome inhibitor, to inhibit the protein degradation by proteases, the protein expression level of VDAC1 decreased by DYNLT1 inhibition was correspondingly increased, indicating that DYNLT1 regulates VDAC1 protein stability through the ubiquitin-proteasome pathway (Fig. 6C and D). To further confirm this conclusion, we overexpressed exogenous HA-VDAC1, His-Ubiquitin and Flag-DYNLT1 in 293T cells to detect the effect of DYNLT1 overexpression on VDAC1 ubiquitination. Our results showed that when exogenous HA-VDAC1 and His-Ubiquitin were transferred, the ubiquitination level of VDAC1 was significantly increased, but after overexpression of DYNLT1, the ubiquitination level of VDAC1 was significantly inhibited, indicating that DYNLT1 promoted the protein stability by inhibiting the ubiquitination of VDAC1 (Fig. 6E). Ubiquitination degradation of proteins is an important degradation mode in the process of protein degradation, and a variety of ubiquitination ligases are involved, including E1, E2 and E3 (Lecker et al. 2006). Among them, E3 ubiquitination ligase can specifically select substrate proteins for ubiquitination modification. Therefore, we wanted to further investigate the E3 ubiquitination ligase regulated by DYNLT1. First, we found that VDAC1 can interact with the E3 ligase Parkin in breast cancer cells (Fig. 6F and G). Parkin, an E3 ligase also known as PRKN, has been reported to interact with mitochondrial outer membrane proteins to regulate mitochondrial functions, such as mitochondrial autophagy (Chen et al. 2022a, b; Harmuth et al. 2022). Then, we knocked down the expression of DYNLT1 to explore how it regulates stability of VDAC1. We found that when DYNLT1 expression was inhibited, the interaction between VDAC1 and the E3 ligase Parkin was further enhanced, suggesting that DYNLT1 could hinder the interaction between VDAC1 and Parkin and thus stabilize the expression of VDAC1 (Fig. 6H and I).

In conclusion, our study demonstrated that DYNLT1 can inhibit the ubiquitination modification of VDAC1 and enhance the protein stability of VDAC1 by inhibiting the interaction between VDAC1 and E3 ligase Parkin, and thereby facilitating biological processes related to ATP levels and mitochondrial permeability in mitochondria and ultimately promoting the development of breast cancer.

## Discussion

Breast cancer is the most common type of cancer in women. It has multiple subtypes, and different subtypes mean different treatments and prognostic outcomes. ER + breast cancer accounts for 70% and endocrine therapy such as tamoxifen is usually used to treat ER + breast cancer patients. However, patients with ER + subtype will have a poor prognosis such as relapse and drug resistance in the later stage (Saatci et al. 2021). Although TNBC accounts for 10–15%, patients with this subtype have a worse prognosis due to the lack of effective targeted drugs (Luo et al. 2022). Here, we demonstrate that DYNLT1 inhibition can effectively inhibit the proliferation, clone formation, migration and invasion of ER + and TNBC cells, suggesting that DYNLT1 can be used as a new therapeutic target for multiple subtypes, providing a new research direction for the treatment of breast cancer.

Voltage-dependent anion channel 1 (VDAC1) is a crucial mitochondrial transporter that controls the flow of ions and respiratory metabolites into and out of mitochondria. Previous research has reported that VDAC1 is a pan-cancer target, which is significantly overexpressed in breast cancer, cervical squamous cell carcinoma, pancreatic cancer, lung adenocarcinoma and melanoma, and the upregulation of VDAC1 is significantly associated with poor prognosis (Wang et al. 2022). DYNLT1 is a new therapeutic target for breast cancer, and the regulatory mechanism between DYNLT1 and VDAC1 in breast cancer has not been reported. However, it has been suggested that DYNLT1 regulates microtubule stability and mitochondrial permeability in hypoxia (Xu et al. 2013). In addition, DYNLT1 stabilizes hypoxia-induced mitochondrial damage by interacting with VDAC1 (Fang et al. 2011), suggesting that the above DYNLT1 related biological processes are involved in the regulation of mitochondrial function. Inspired by GO analysis, we verified that DYNLT1 was indeed involved in ATP accumulation and the regulation of mitochondrial membrane potential. Furthermore, we demonstrated that DYNLT1 can interact with VDAC1 to promote the protein stability of VDAC1 through antagonizing the interaction of E3 ligase Parkin with VDAC1, inhibiting the ubiquitination degradation of VDAC1 and stabilizing its expression, which explained the mechanism of DYNLT1 in regulating mitochondrial metabolism. To date, no drugs targeting DYNLT1 have been developed, while VBIT-12, a specific small molecule inhibitor targeting VDAC1, exists to improve amyotrophic lateral sclerosis (ALS) in mice (Shteinfer-Kuzmine et al. 2022), alleviate acute liver injury in mice (Niu et al. 2022), and alleviate inflammatory bowel disease due to mitochondrial dysfunction in mice (Verma et al. 2022), and the application of VBIT-12 in cancer has not reported. Therefore, whether inhibitors targeting DYNLT1 can be combined with VDAC1 inhibitors to achieve a therapeutic effect in breast cancer remains to be further explored.

## Conclusions

In summary, this study demonstrates that DYNLT1 promotes mitochondrial metabolism to fuel breast cancer development by inhibiting Parkin-mediated ubiquitination degradation of VDAC1. Our data suggest that mitochondrial metabolism can be exploited by targeting the DYNLT1-Parkin-VDAC1 axis to further improve the ability of metabolic inhibitors to suppress breast cancers with limited treatment options, such as TNBC, in the future.

## Electronic supplementary material

Below is the link to the electronic supplementary material.

Supplementary Material

## Data Availability

The datasets used and analyzed during the current study are available from the corresponding author on reasonable request.

## Abbreviations

DYNLT1: Dynein Light Chain Tctex-Type 1
VDAC1: Voltage-dependent anion channel 1
TNBC: Triple-negative breast cancer
ER: Estrogen receptor
PR: Progesterone receptor
HER2: Human epidermal growth factor receptor 2
PDH: Pyruvate dehydrogenase
OXPHOS: Oxidative phosphorylation
GO: Gene ontology
KEGG: Kyoto Encyclopedia of Genes and Genomes
