# Supplementary material for: DYNLT1 promotes mitochondrial metabolism to fuel breast cancer development by inhibiting ubiquitination degradation of VDAC1
Source: Mol Med. 2023 Jun 6;29:72. doi: 10.1186/s10020-023-00663-0 (PMC10245490; doi:10.1186/s10020-023-00663-0)
Supplement: Supplementary file 1 — Supplementary Material [file 10020_2023_663_MOESM1_ESM.zip › Supplementary files_10020_2023_663_Article/Supplementary Information.pdf]

Supplementary Information for

DYNLT1 promotes mitochondrial metabolism to fuel breast cancer development by inhibiting ubiquitination degradation of VDAC1

Authors: Ling Huang[1,2], Bo Wei[1,2], Yuran Zhao[1,2], Xue Gong[3,*], Liming Chen[1,2,*]

Correspondence to: Xue Gong, gongxue@njmu.edu.cn; Liming Chen, chenliming1981@njnu.edu.cn.

This file includes:

Supplementary Fig. 1 to 3

Other Supplementary Materials for this manuscript include the following:

Table S1: List of top 500 genes significantly associated with DYNLT1.

Fig. S1

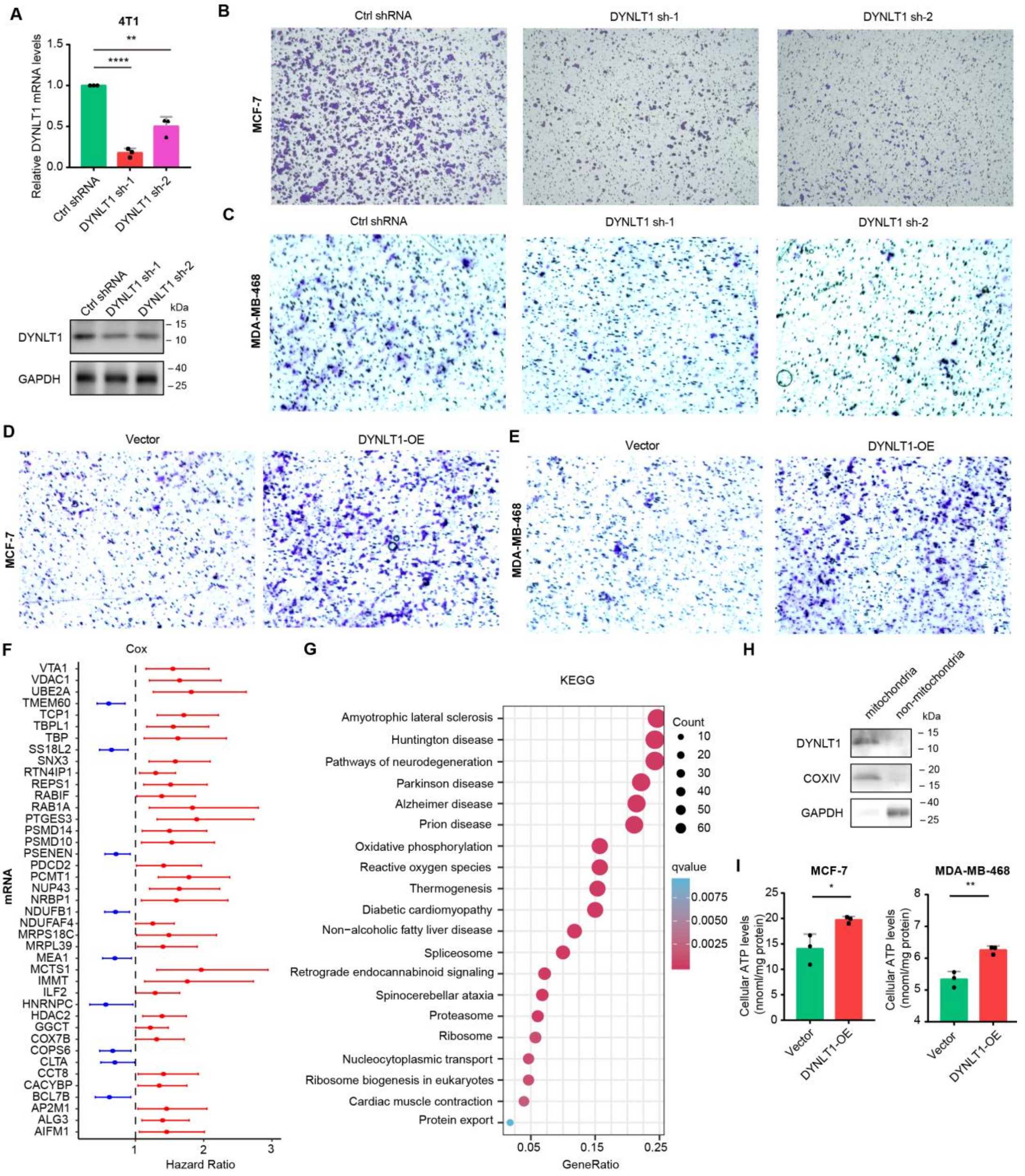

Fig. S1. DYNLT1 affects mitochondrial metabolism in breast cancer.

A RT-qPCR (up) and WB (down) results showed the successful construction of stable DYNLT1 knockdown 4T1 cells. **B-C** The invasion of stable DYNLT1 knockdown MCF-7 (B) or MDA-MB-468 (C) breast cancer cells with transwell chamber containing matrigel. **D-E** The invasion after overexpression of DYNLT1 in MCF-7 (D) or MDA-MB-468 (E) breast cancer cells with transwell chamber containing matrigel.

F Forest plot showing prognostic analysis of the top 500 genes significantly associated with DYNLT1. Hazard ratio of more than 1 indicates that the gene is a risk factor for survival, while a Hazard ratio of less than 1 indicates that the gene is a protective factor. **G** KEGG enrichment analysis of top-500 genes significantly associated with DYNLT1. **H** Protein levels of DYNLT1, COXIV and GAPDH in enriched mitochondria fraction or non enriched mitochondrial fraction. COXIV represents the positive marker in mitochondria and GAPDH represents the positive marker in non-mitochondria. **I** The cellular ATP levels after overexpression of DYNLT1 in MCF-7 (left) or MDA-MB-468 (right) breast cancer cells. *, $p<0.05$; **, $p<0.01$; ****, $p<0.0001$.

Fig. S2

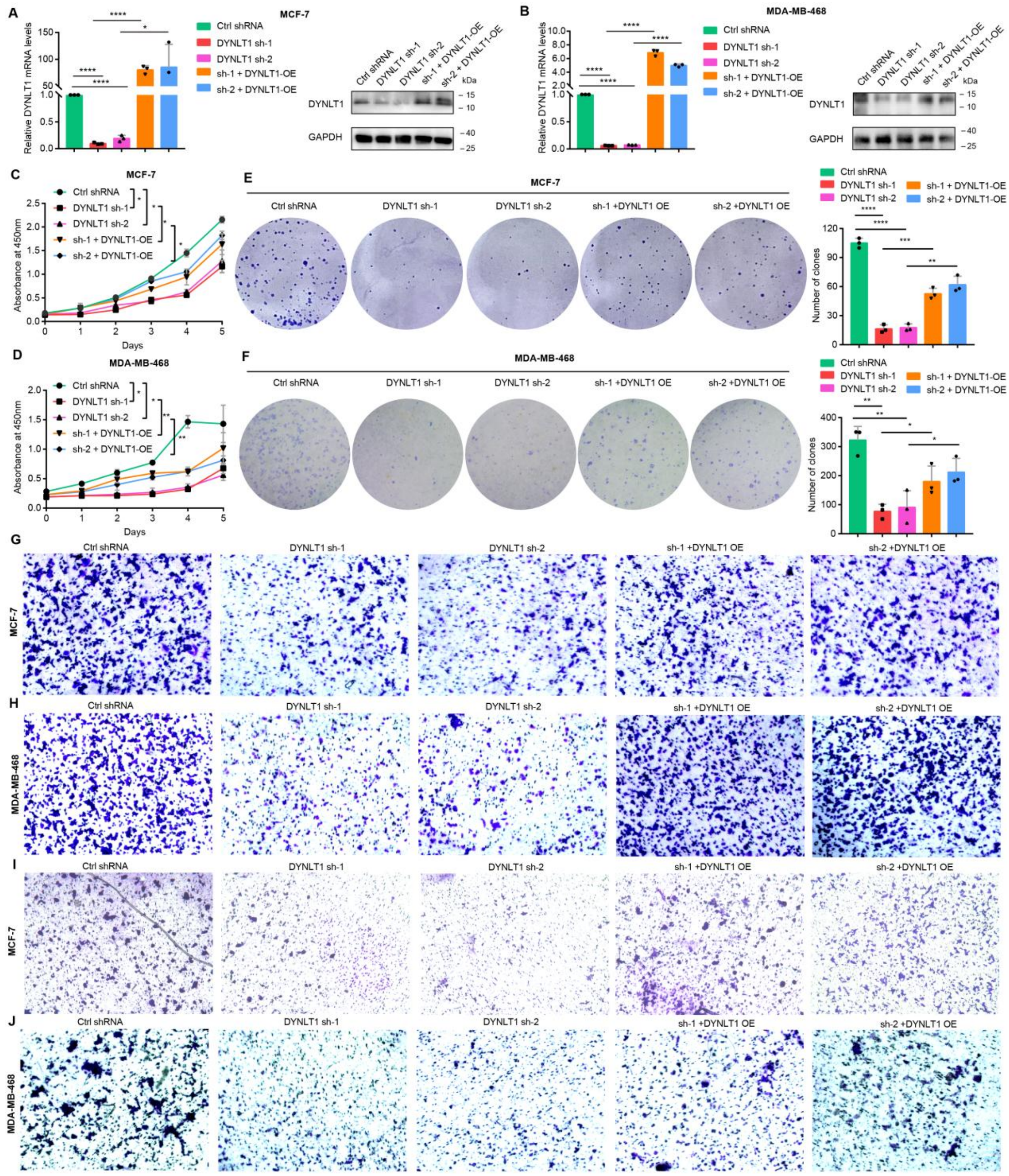

Fig. S2. DYNLT1 promotes the proliferation, clone formation, migration and invasion in breast cancer cells.

A-B RT-qPCR and Western blot showed the expression of DYNLT1 and GAPDH with indicated treatment in MCF-7 (A) and MDA-MB-468 (B) cells. **C-D** The proliferation of MCF-7 (C) and MDA-MB-468 (D) cells with indicated treatment by

CCK8 assay. **E-F** The ability of clone formation of MCF-7 (E) and MDA-MB-468 (F) cells with indicated treatment. The graph on the right shows the statistics of the number of clones. **G-H** The migration of MCF-7 (C) and MDA-MB-468 (D) cells with indicated treatment. **I-J** The invasion of MCF-7 (I) and MDA-MB-468 (J) cells with indicated treatment with transwell chamber containing matrigel. *, $p<0.05$; **, $p<0.01$; ****, $p<0.0001$; ns, no significance. All experiments were performed at least three replicates.

Fig. S3

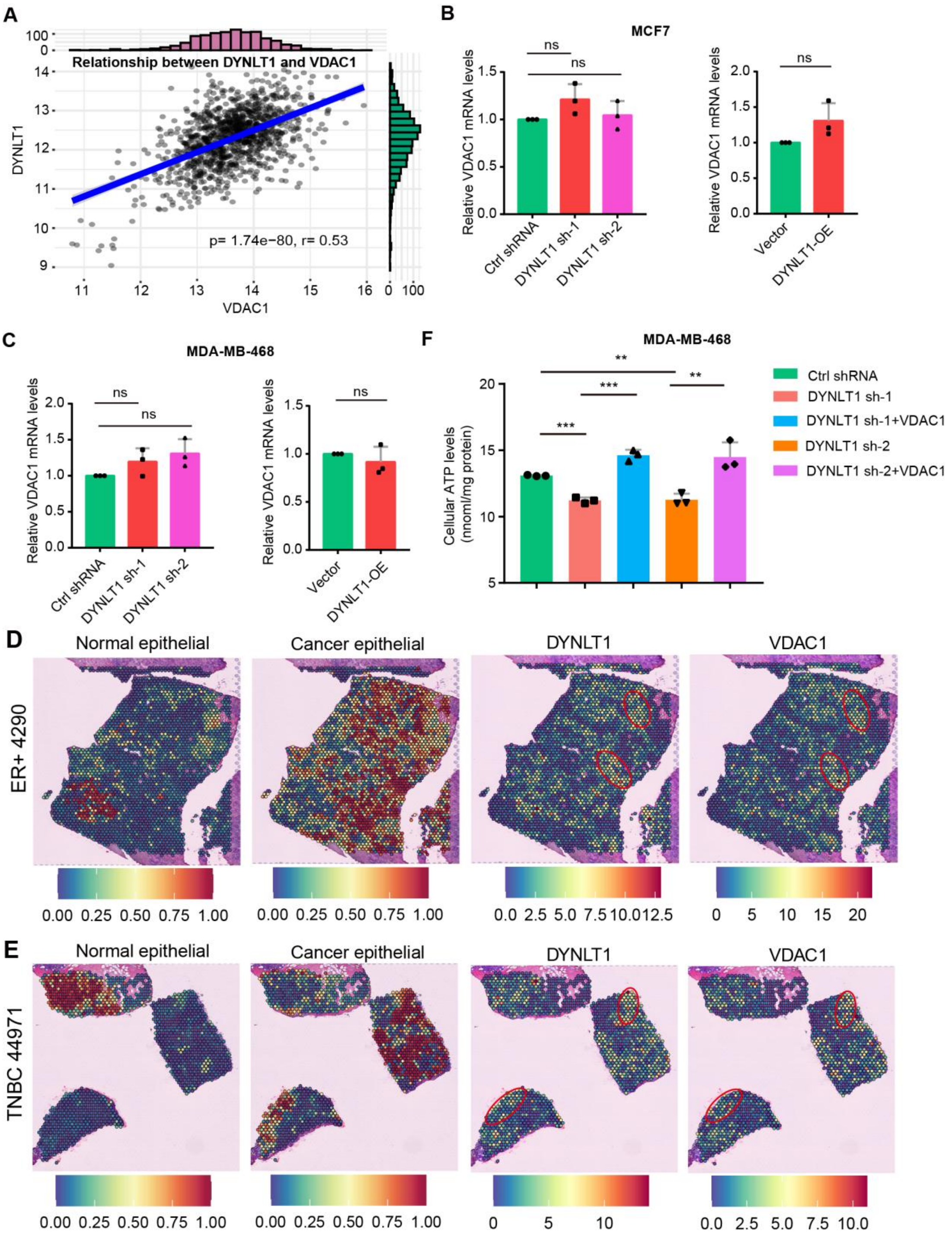

Fig. S3. DYNLT1 and VDAC1 are co-expressed and co-localized in breast cancer.

A Correlation analysis of DYNLT1 and VDAC1. **B** Relative VDAC1 mRNA levels after DYNLT1 knockdown (left) or overexpression (right) in MCF-7 cells. **C** Relative VDAC1 mRNA levels after DYNLT1 knockdown (left) or overexpression (right) in MDA-MB-468 cells. **D-E** Spatial transcriptome data of patients with ER+ and TNBC subtypes of breast cancer. The red circle marks the adjacent area of the spatial position of DYNLT1 and VDAC1. **F** The cellular ATP levels with indicated treatment in MCF-7 cells. *, $p<0.05$; **, $p<0.01$; ***, $p<0.001$; ns, no significance.
